# The association between sleep disturbances and tooth loss among post-stroke patients

**DOI:** 10.1590/0004-282X-ANP-2020-0368

**Published:** 2022-03-15

**Authors:** Eliana Lottenberg VAGO, Cristina FRANGE, Giuliano DA PAZ OLIVEIRA, Maria Ligia JULIANO, Marco Antônio MACHADO, Fernando Morgadinho Santos COELHO

**Affiliations:** 1 Universidade Federal de São Paulo, Departamento de Neurologia e Neurocirurgia, São Paulo SP, Brazil. Universidade Federal de São Paulo Departamento de Neurologia e Neurocirurgia São Paulo SP Brazil; 2 Universidade Federal de São Paulo, Departamento de Psicobiologia, São Paulo SP, Brazil. Universidade Federal de São Paulo Departamento de Psicobiologia São Paulo SP Brazil

**Keywords:** Stroke, Sleep, Sleep Apnea, Obstructive, Jaw, Edentulous, Acidente Vascular Cerebral, Sono, Apneia Obstrutiva do Sono, Arcada Edêntula

## Abstract

**Background:**

Loss of teeth has been associated with neurological and sleep disorders. It is considered to be a predictor of stroke and leads to modifications of airway patency and predisposition to obstructive sleep apnea.

**Objective:**

To investigate sleep quality, risk of obstructive sleep apnea and excessive sleepiness among post-stroke patients with tooth loss attending the Neurovascular Clinic of the Federal University of São Paulo.

**Methods:**

The prevalence rates of different types of stroke were assessed among 130 patients with different degrees of tooth loss, along with the presence of sleep disturbances, risk of obstructive sleep apnea and excessive daytime sleepiness.

**Results:**

The prevalence of ischemic stroke was 94.6%, with either no significant disability or slight disability. Our sample had poor sleep quality, and a high risk of obstructive sleep apnea, but without excessive daytime sleepiness. Half of our sample had lost between 9 and 31 teeth, and more than 25% had edentulism. The majority used full removable dental prostheses, and more than half of these individuals slept without removing the prosthesis.

**Conclusions:**

We found high prevalence of poor sleep quality and high risk of obstructive sleep apnea among post-stroke patients with tooth loss. This indicates the need for further studies on treating and preventing sleep disturbances in stroke patients with tooth loss.

## RESUMO

**Antecedentes:**

A perda de dentes tem sido associada a distúrbios neurológicos e do sono. É considerada um preditor de acidente vascular cerebral (AVC), com modificações na permeabilidade das vias aéreas e predisposição à apneia obstrutiva do sono.

**Objetivo:**

Investigar a qualidade do sono, o risco de apneia obstrutiva do sono e a sonolência excessiva em pacientes pós-AVC com perda dentária, atendidos na Clínica Neurovascular da Universidade Federal de São Paulo.

**Métodos:**

O estudo avaliou a prevalência de diferentes tipos de AVC em 130 pacientes com diferentes graus de perda dentária e a presença de distúrbios do sono, risco de apneia obstrutiva do sono e sonolência excessiva.

**Resultados:**

A prevalência de AVC isquêmico foi de 94,6%, sem deficiência significativa ou deficiência leve. Nossa amostra tinha má qualidade de sono e alto risco de apneia obstrutiva do sono, sem sonolência diurna excessiva. Metade de nossa amostra perdeu entre nove e 31 dentes, e mais de 25% tiveram edentulismo. A maioria usava próteses dentárias totalmente removíveis e, desses pacientes, mais da metade dormia com elas.

**Conclusões:**

Encontramos alta prevalência de má qualidade do sono e alto risco de apneia obstrutiva do sono em pacientes pós-AVC com perda dentária. Isso indica a necessidade de mais estudos sobre o tratamento e a prevenção de distúrbios do sono em pacientes com AVC e perda dentária.

## INTRODUCTION

Stroke is one of the main causes of disability and death in many regions of Brazil^1^, and has been associated with tooth loss. This suggests that improving the periodontal condition of the general population could reduce overall mortality^2^. Moreover, tooth loss has been shown to be a predictor of stroke and cerebral white matter changes. It is an easy-to-assess and cost-effective indicator of periodontitis, a chronic inflammatory condition that is especially common in late life, in which the associated bacteremia can cause vascular damage^3^.

Stroke patients may present sensory-motor, musculoskeletal, perceptual and cognitive sequelae, as well as sleep disturbances. However, although sleep problems are common in patients with stroke, it is not yet known whether they appear before the event or are exacerbated by it^4^. The relationship between stroke and sleep disturbances may be both causal and bidirectional^5^. On the one hand, it has been shown that sleep disturbances such as obstructive sleep apnea (OSA) are an independent risk factor for stroke^6^. On the other hand, OSA, excessive daytime sleepiness (EDS), poor sleep quality, complaints of non-restorative sleep and restless legs syndrome (RLS) occur frequently after stroke^7^.

High prevalence of respiratory sleep disorders has been reported among patients with stroke. Up to 40% of individuals with chronic stroke and 70% of those with acute stroke present these disorders^8^. Sleep-disordered breathing has been shown to have a negative impact on sleep quality after stroke, and it increases both the risk of another stroke and the risk of other cardiovascular events. OSA is associated with increased risks of diabetes, obesity and cardiovascular diseases such as hypertension, along with potentiating arrhythmias and embolisms^9^.

Loss of teeth has been shown to be an independent risk factor for OSA. Each missing tooth increases the risk for OSA, such that the risk is 25% higher among those who have lost 5 to 8 teeth, 36% higher among those who have lost 9 to 31 teeth and 61% higher among those who have lost all their teeth (edentulism)^10^. Edentulism *per se* can lead to morphological modifications in the orofacial region (and of course, periodontitis) that negatively impact airway patency, thus predisposing individuals to OSA through restricting or obstructing the upper airway^11^. A combination of upper-airway anatomical abnormalities, imbalances in neural activation mechanisms and structural changes (retrognathia, posterior pharyngeal walls, a larger and/or softer tongue and palate, and tooth loss) have been implicated in the pathogenesis of OSA.

To the best of our knowledge, the overlap between tooth loss, sleep disturbances and stroke has not as yet been investigated. We hypothesized that after stroke, tooth loss may negatively influence the prevalence of OSA and symptoms such as EDS and poor sleep quality, thereby resulting in higher levels of disabilities. Therefore, the objectives of this investigation were to investigate EDS, poor sleep quality and the risk of OSA, and to assess whether there was any association between these sleep-related factors and tooth loss among post-stroke patients.

## METHODS

### Study design and study population

A total of 130 patients with tooth loss who had experienced a stroke were recruited to participate in this study between March 2016 and December 2017. The participants attended the Neurovascular Outpatient Clinic of the Universidade Federal de São Paulo (UNIFESP). The study protocol was approved by the Institutional Research Ethics Committee of UNIFESP. This was a cross-sectional observational study. Assessments were made by completing forms and questionnaires with the participants. Informed consent was obtained from all patients.

The inclusion criteria were the following: age ≥18 years old and having had an ischemic or hemorrhagic stroke, as verified from the medical records.

The exclusion criteria comprised: psychiatric illness (because of the possibility that prescribed medication might interfere with sleep); severe cognitive impairment; aphasia; and use of sedative or hypnotic medications. Thus, 14 participants were excluded: five due to aphasia, seven patients who did not complete the questionnaires and two due to dementia.

### Data collection and clinical assessments

Data were gathered from the participants’ medical records, clinical measurements and completed questionnaires, including the following information:

- Sociodemographic and clinical data: age, sex, body mass index (BMI) [calculated through the formula weight (kg)/height^2^ (m^2^)], neck circumference measurement, smoking status, presence of systemic arterial hypertension (SAH; defined as systolic blood pressure of ≥140 mmHg or diastolic blood pressure of ≥90 mmHg, or regular use of antihypertensive medication), diabetes mellitus (DM; defined as fasting blood glucose concentration of 126 mg/dl or higher, or current use of antidiabetic medication) and dyslipidemia (DLP; defined in accordance with the Medical Guidelines for Clinical Practice of the American Association of Clinical Endocrinologists);
- Stroke data: quantitative measurements of stroke-related neurological deficit or stroke severity were assessed in accordance with the National Institutes of Health Stroke Scale (NIHSS)^12^, which ranges from 0 to 42, such that higher scores indicate greater severity stroke; the etiological classification of stroke was made in accordance with the Trial of Org 10172 in Acute Stroke Treatment (TOAST)^13^.
- The degree of disability or dependence within the participants’ daily activities was measured in accordance with the modified Rankin Scale (mRS), which ranges from 0 to 6, such that 0 describes participants without symptoms; grade 1, participants without significant disability despite symptoms; grade 2, slight disability; grade 3, moderate disability; grade 4, moderately severe disability; grade 5, severe disability; and grade 6, death.
- Sleep measurements: Sleep quality was assessed using the Pittsburgh Sleep Quality Index (PSQI). A PSQI of ≤5 indicates good sleep quality, while >5 is associated with poor sleep quality and >10 indicates sleep disturbances. The risk of OSA was measured using the STOP-Bang questionnaire^14^, in which the scores range from 0-8, such that 0-2 represents low risk of OSA; 3-4, intermediate risk; and 5-8, high risk. Excessive diurnal sleepiness was evaluated using the Epworth Sleepiness Scale (ESS), in which the scores range from 0 to 24, such that 0-9 indicates “no sleepiness symptoms”, while >9 may be “suggestive of daytime sleepiness”.
- Tooth loss and occlusal contacts: a questionnaire on buccal health was completed, including the use of dentures; and a dental examination was conducted on each participant to collect anatomical characteristics such as the number of teeth and the condition of the palate, uvula and tongue.
- The modified Mallampati classification was used for airway classification. This is scored from 1 to 4 based on the anatomical features of the airway with the mouth open and tongue protruded maximally without phonation: grade I - tonsils, pillars and soft palate are all clearly visible; grade II - uvula, pillars and upper pole are visible; grade III - only part of the soft palate is visible, and the uvula is somewhat hidden; and grade IV - only the hard palate is visible^15^^,^^16^^,^^17^. The tonsils were classified using the Brodsky Tonsil Scale (BTS): grade 0 - previous tonsillectomy; grade 1 - tonsils were hidden in the pillars; grade 2 - tonsils were beyond the anterior pillar and occupied between 25 and 50% of the pharyngeal space; grade 3 - tonsils were beyond the pillars but not to the middle and occupied >50% and up to 75% of the pharyngeal space; grade 4 - tonsils occupied >75% of the pharyngeal space. Bite was categorized as one of three different types, in relation to the position of the first molars and the way in which the upper molars fit together with the lower ones (types I, II and III)^18^.

### Statistical analysis

Data analysis was performed using *Statistical Package for the Social Sciences* version 21.0 (SPSS Inc., Chicago, Illinois, USA). The results were presented as mean±SD or median and interquartile ranges (IQR, 25-75), and percentage, depending on the normality of the data. Student’s *t*-test or ANOVA was used for comparison of means, and the chi-square and Fisher tests were used to compare proportions. Correlations between stroke and sleep variables and between stroke and anatomical characteristics were made using Spearman’s correlation test. P values of less than 0.05 were considered significant.

## RESULTS

Out of the 130 participants in the study, 52.3% were men. The participants’ mean age was 59.7 (±12.5) years; mean BMI, 26.4 (±5.0 kg/m^2^); and neck circumference, 46.4 (±4.3 cm). The prevalence rates of hypertension, DM and smoking were 103 (79.2%), 49 (37.7%) and 23 (17.7%), respectively.

The prevalence of ischemic stroke was 94.6% (123 patients) and hemorrhagic stroke, 5.4% (7 patients). The median NIHSS score was 2.0 (IQR 3.0), and the median mRS score was 2.0 (IQR 2.0). Among the 123 patients diagnosed with ischemic stroke, according to the TOAST classification, 29.27% had ischemic stroke of other determined etiology, 23.58% cardioembolism, 21.14% large-artery atherosclerosis, 17.7% small-vessel occlusion and 8.9% stroke of undetermined etiology.

Regarding sleep measurements, the mean score for sleep quality (PSQI) was

7.13 (±3.84), thus characterizing poor sleep quality. The participants were considered to be at high risk of OSA, according to the results obtained from the STOP-Bang questionnaire (4.11±1.57). The mean score for excessive daytime sleepiness was 8.16 (±5.22), thus indicating that there was no diurnal somnolence in this sample. Among our sample, 10.8% (14 participants) had a diagnosis of RLS.

Regarding the number of missing teeth observed, 48.9% of our sample had lost between 9 and 31 teeth, and 26.2% were edentulous. The percentage of patients using complete, removable dentures was 60.8%, and half of these patients habitually slept without removing the dentures. Ogival palate was present in 10.8% of the sample and web palate in 6.9%. Mallampati scores of III and IV were noted in 71.5%, tonsils grade I were present in 89.2%, and 82.3% had normal bite (Table 1).

**Table 1.** Characteristics of oral cavity, oropharynx, face and bite among patients with tooth loss after stroke (n, %).

Hard palate	Normal=14 (10.77%)	Oval=116 (89.33%)			
Soft palate	Normal=16 (12.30%)	Web=13 (10%)	Thick=7 (5.38%)	Long=94 (72.32%)	
Uvula	Normal=72 (55.38%)	Long=3 (2.31%)	Thick=32 (23.02%)	Surgery=22 (15.83%)	
Tongue	Normal=108 (77.77%)	Marked by teeth=20 (15.38%)	Hypotonic=1 (0.72%)		
Mallampati	Score I=4 (3.08%)	Score II=13 (9.35%)	Score III=19 (14.62%)	Score IV=93 (71.54%)	
Tonsils	Grade 1=5 (3.85%)	Grade 2=116 (89.23%)	Grade 3=6 (4.62%)	Grade 4=2 (0.01%)	
Face profile	Normal=37 (28.46%)	Short=77 (59.23%)	Long=15 (11.54%)		
Bite	Normal=107 (82.3%)	Open=1 (0.77%)	Deep=5 (3.85%)	Crossbite=4 (3.08%)	End to End=9 (6.92%)

Stroke severity was correlated with Mallampati scores (rho=0.174; p=0.048) and negatively correlated with BTS (r=-0.199; p=0.023). There were also significant positive correlations between increased daytime sleepiness and disability (r=0.236; p=0.007), BTS (r=0.172; p=0.05) and number of teeth between 5 and 8 (r=0.227; p=0.009). Poor sleep quality was positively correlated with Mallampati scores (r=0.2; p=0.023). Given that the correlations were all weak, we were unable to perform a regression analysis.

## DISCUSSION

This investigation on the associations between tooth loss and sleep disturbances among patients after stroke found that almost 95% of the sample had had an ischemic stroke of low severity, and there was non-significant presence of slight disability. This reflected the population of the outpatient clinic in which we recruited the participants. This finding was in line with the evidence in the literature, as 87% of stroke cases are ischemic and 13% hemorrhagic^19^^,^^20^. In addition, this finding is in agreement with the low levels of disabilities found in our sample, which is more commonly observed after ischemic events^21^, and which indicates a strong probability of good recovery from the stroke. The majority of our sample comprised older men (≥40 years) who smoked and presented comorbid hypertension and diabetes. These are overlapping risk factors that predispose to both stroke and OSA. Older age, hypertension and smoking are well-known risk factors for stroke^22^. There is evidence that after the first cerebrovascular event, patients usually do not change their habits and thus have recurrent stroke^23^. High prevalence of embolic stroke was found in our sample, and this can be characterized by different phenotypes, depending on each population^24^.

Despite the low severity of stroke in our sample, we found high risk of OSA and a self-perception of poor sleep quality. These factors can have a tremendously negative impact on stroke recovery, thereby increasing the risk of recurrent events. OSA (and its larger umbrella condition, namely sleep-disordered breathing, which includes central sleep apnea, sleep-related hypoventilation and Cheyne-Stokes breathing) is suspected to be present in 50-70% of patients after stroke^25^. In another investigation, our group found an association between poor sleep quality and increased risk of sleep-disordered breathing^26^.

In this regard, sleep and stroke are interrelated, given that pre-existing sleep disturbances increase the risk factor for stroke. Patients with untreated OSA tend to have heightened sympathetic activity and autonomic dysregulation, and acute strokes can lead to the development of sleep-disordered breathing. Several studies comparing patients with and without OSA have found a 2 to 4.5-fold greater independent risk of a first-ever event of ischemic stroke among patients with OSA, which suggests that OSA may constitute a pre-existing condition rather than being a consequence of acute ischemic stroke. Furthermore, the risk of suffering a recurrent event may be noticeably higher among patients with OSA after stroke^27^. Treatment of sleep disorders such as OSA after stroke onset can enhance functional recovery, especially with regard to depression and sedentarism, improved concentration and attention and increased ability to perform activities of daily living.

Therefore, early diagnosis and treatment of OSA should reduce the risk of stroke. Polysomnographic (PSG) examinations are considered to be the golden standard for OSA diagnosis, but the prohibitive cost of the test and long waiting lists limit widespread access to it. Sleep specialists have proposed use of the STOP- Bang questionnaire as an alternative, more accessible screening tool, at least in the initial evaluation. The STOP-Bang questionnaire has high sensitivity (SE=95%) for identifying people at higher risk of OSA and, although its specificity is low (SP=16%), it is a simple and cost-effective instrument. In our sample, the risk of OSA was high among patients with tooth loss after stroke^28^.

Patients with tooth loss are at higher risk of developing OSA, given that morphological changes in the upper airways can cause restriction and/or obstruction, thus leading to OSA and a cascade of events, such as EDS, poor sleep quality and concomitant sleep disturbances, such as restless legs syndrome^10^. Poor dental conditions (periodontal disease) and/or loss of teeth impact quality of life and affect the type of food eaten and its preparation, and this has been shown to be strongly associated with a myriad of diseases^29^. Periodontal diseases, which are one of the major causes of tooth loss, have been associated with OSA^30^.

In our analysis on the oral cavity, we found Mallampati scores of III and IV in more than 70% of our sample, along with an elongated soft palate, which increases pharyngeal collapse: these are common findings in OSA cases. Anatomical and functional changes to craniofacial structures, such as a retrognathic jaw, diminished posterior pharyngeal wall, tooth loss and large soft tongue and palate have been implicated in the pathogenesis of OSA^31^. Indeed, some investigations have shown that tooth loss has the capacity to change the position of the mandible, decrease the vertical dimension of occlusion, impair functioning of the oropharyngeal musculature (e.g. loss of tone in the soft palate and pharynx and occurrence of macroglossia) and change the position of the hyoid bone. Changes to the soft palate, hard palate and mandibular position are important risk factors for OSA. Patients with an elongated soft palate have been shown to have higher rates of OSA, as assessed through PSG^32^. Another study showed that 31% of the population with tooth loss was identified as presenting high risk of OSA; however, until now, no investigations had been conducted among post-stroke participants^33^. Tooth loss plays an especially important role in terms of the respiratory process, body balance and general health of the stomatognathic system. Nevertheless, the exact mechanisms underlying this relationship remain unknown.

Currently, there is no consensus in the literature regarding the use of dentures by patients during sleep, or regarding their benefit in relation to OSA. Almost half of our sample had lost between 9 and 31 teeth, and more than a quarter had edentulism. More than 60% of our sample reported that they continued to use their dentures during the night. Several investigations have reported that wearing dentures at night can decrease the severity of OSA^34^. Patients with tooth loss who sleep with their dentures in do not seem to show any objective changes in sleep (i.e. with regard to polysomnographic parameters).

The apnea-hypopnea index (AHI) is a measurement that forms part of PSG examinations. It is considered in making OSA diagnoses since it represents the number of apneas and hypopneas per hour of sleep. In a meta-analysis, no significant differences in the AHI of individuals using a dental prosthesis during sleep were found. This suggested that objective measurements showed that use of a dental prothesis failed to diminish AHI, and thus the severity of OSA^35^. However, contrary to the findings of that meta-analysis, another study reported that use of a dental prosthesis overnight by patients with tooth loss increased the AHI, thereby suggesting that it would be advisable to remove the denture before going to bed^36^. These contradictory findings may have been due to heterogeneity among the studies, and differences in measuring OSA.

In our sample, most of the participants slept without removing their dentures, but our results cannot be compared with the results from other studies, given that there is a lack of investigations on dental protheses after stroke. In clinical practice, we have seen that use of dentures can prevent OSA, but each specific case needs to be considered by a dentist specializing in sleep medicine. This is because a number of drawbacks exist regarding overnight use of dentures, such as resorption of the alveolar bone in the support area for the prosthesis, chronic inflammatory alterations in the patient and changes to the vertical dimension of occlusion, which can cause tension in the temporomandibular joint, with poor adaptation^32^. Therefore, additional studies are urgently required in order to evaluate the effects of tooth loss and use of dentures, in larger samples of patients.

Interestingly, a recent case report discussed the case of a patient with an oxygen desaturation index of almost 21 at baseline, who began to use removable dentures during sleep to prevent upper airway collapses. The oxygen desaturation index measures the number of times per hour that oxygen saturation decreases. This index dropped to 12.6 through use of a denture prothesis. The removable lower total prosthesis was then converted into a prosthesis in which retention was supported by means of two implants (overdenture). After 6 months, the oxygen desaturation index was 7.8. In this case, use of a total prosthesis improved this patient’s respiratory stability during sleep^37^.

Our sample did not present EDS, which reflected a situation of no post-stroke sleepiness, thus differing from the reports from other previous investigations. EDS may be caused by OSA or depression and is correlated with negative health outcomes after stroke. Although EDS is very common in OSA, the association between them has been reported to be weak in middle-aged adults^38^.

Poor sleep quality is intrinsically related to OSA and other sleep disturbances, but recent investigations on tooth loss have found a 4% increase in the odds of having short sleep duration (i.e. less than 6 h/night) for each subsequent tooth loss. In individuals with less than 20 teeth, short sleep duration is also more prevalent^39^. In addition, loss of the teeth might be attributed to emotional distress and psychological problems, or to orofacial pain and temporomandibular disorders^40^.

Our investigation had some limitations. Its cross-sectional nature precluded inferences about temporal sequence or causality. Patients with no sleep disturbances at the time of the evaluation could have developed sleep disturbances later, and evaluations of such occurrences would require a longitudinal study. In addition, this was a single-center investigation, and we did not perform any PSG examination to assess the AHI of our sample. We excluded patients with psychiatric illnesses who were undergoing treatment. After stroke, depressive episodes are common, and the medications used in treatments for these conditions could modify sleep quality and EDS. Another limitation was that airway volume was not measured during sleep. The contribution of tooth loss to airway obstruction may have been amplified through muscle hypotonia/atonia during the sleep. Nevertheless, despite these limitations, this was the first study to have evaluated the associations between tooth loss and sleep quality, risk of OSA and excessive daytime sleepiness among stroke patients in Brazil.

In conclusion, despite the complex and sometimes bi-directional relationships between tooth loss, sleep disturbances and stroke, we found high prevalences of poor sleep quality and risk of obstructive sleep apnea among patients with tooth loss after stroke. There is a paucity of effective evidence-based therapeutic strategies for OSA patients with tooth loss after stroke, and our study highlights the need for further randomized clinical trials on treatments for improving clinical management.
